# Aspergillus Myosin-V Supports Polarized Growth in the Absence of Microtubule-Based Transport

**DOI:** 10.1371/journal.pone.0028575

**Published:** 2011-12-14

**Authors:** Jun Zhang, Kaeling Tan, Xufeng Wu, Guifang Chen, Jinjin Sun, Samara L. Reck-Peterson, John A. Hammer, Xin Xiang

**Affiliations:** 1 Department of Biochemistry and Molecular Biology, Uniformed Services University of the Health Sciences, F. Edward Hébert School of Medicine, Bethesda, Maryland, United States of America; 2 Department of Cell Biology, Harvard Medical School, Boston, Massachusetts, United States of America; 3 Laboratory of Cell Biology, National Heart, Lung and Blood Institute, National Institutes of Health, Bethesda, Maryland, United States of America; 4 River Hill High School, Clarksville, Maryland, United States of America; Universidade de Sao Paulo, Brazil

## Abstract

In the filamentous fungus *Aspergillus nidulans*, both microtubules and actin filaments are important for polarized growth at the hyphal tip. Less clear is how different microtubule-based and actin-based motors work together to support this growth. Here we examined the role of myosin-V (MYOV) in hyphal growth. MYOV-depleted cells form elongated hyphae, but the rate of hyphal elongation is significantly reduced. In addition, although wild type cells without microtubules still undergo polarized growth, microtubule disassembly abolishes polarized growth in MYOV-depleted cells. Thus, MYOV is essential for polarized growth in the absence of microtubules. Moreover, while a triple kinesin null mutant lacking kinesin-1 (KINA) and two kinesin-3s (UNCA and UNCB) undergoes hyphal elongation and forms a colony, depleting MYOV in this triple mutant results in lethality due to a severe defect in polarized growth. These results argue that MYOV, through its ability to transport secretory cargo, can support a significant amount of polarized hyphal tip growth in the absence of any microtubule-based transport. Finally, our genetic analyses also indicate that KINA (kinesin-1) rather than UNCA (kinesin-3) is the major kinesin motor that supports polarized growth in the absence of MYOV.

## Introduction

Type V myosins have been implicated in organelle transport in numerous organisms [1]. In the budding yeast *Saccharomyces cerevisiae*, where cellular transport of vesicles depends solely on the actin cytoskeleton, class V myosins transport most if not all of the cell's organelles, including late Golgi elements, post-Golgi secretory vesicles, peroxisomes, mitochondria, and the endoplasmic reticulum [2]–[7]. In higher eukaryotic cells, where both microtubules and the actin cytoskeleton participate in the intracellular transport of organelles and vesicles, microtubules are used for long-distance transport, while actin filaments are used for short-range transport in the cell periphery [8]–[11]. For example, melanosomes in mouse melanocytes are first transported along microtubules out the cells dendrites, where they are then captured by myosin-V at dendritic tips [8]. Similarly, in mouse cerebellar Purkinje neurons, the endoplasmic reticulum (ER) is first distributed throughout dendrites by microtubule motors, and then transported into dendritic spines by myosin-Va [9]. That said, the extent to which myosin-V acts as a cargo transporter as opposed to a dynamic tether to simply grab cargo following its transport to the microtubule plus end by kinesins, remains a point of intense debate [11], [12]. While the recent characterization of myosin Va-dependent ER transport in Purkinje neurons provided strong support for the idea that myosin-V functions as a point-to-point cargo transporter [9], [12], the extent to which this is true in other cellular contexts remains to be seen.

Filamentous fungi, in contrast to budding yeast, use both microtubules and actin filaments for the transport of cargo that supports polarized growth at the hyphal tip [13]–[18]. Thus, filamentous fungi are well suited for studying how microtubule- and actin-based transport systems are coordinated. Here we used the filamentous fungus *Aspergillus nidulans*, a major fungal model organism [19], to study the function of myosin-V and to ask whether coordination between actin and microtubule tracks is a necessary component of myosin-V-based transport. Our current results indicate that myosin-V in *A. nidulans* is able to support polarized growth on its own, i.e. in the absence of microtubule-based transport, which supports the idea that myosin-V is able to function as a cargo transporter. In addition, we show that in the absence of myosin-V, the kinesin-1 KINA is more important than the kinesin-3 UNCA in supporting hyphal tip growth.

## Results

### Construction of the conditional null mutant of MYOV

The genome of *A. nidulans* contains only one myosin-V heavy chain homolog, which is encoded by the gene An8862 (called “*myoV” here*) [20]. This *myoV* gene was identified via blast search against the *A. nidulans* database using the amino acid sequence of the mouse myosin-Va heavy chain (the product of the *dilute* locus) as a query. As expected, *A. nidulans* MYOV shows significant sequence similarity throughout its N-terminal motor domain with myosin-Vs from other species (data not shown). More importantly, its C-terminal cargo-binding domain is clearly homologous to that of Myo2p in budding yeast [21], myo5 in *U. maydis* [17] and myosin-Va in mouse [22] (Figure S1), confirming that MYOV is a true myosin-V ortholog.

**Figure 1:**
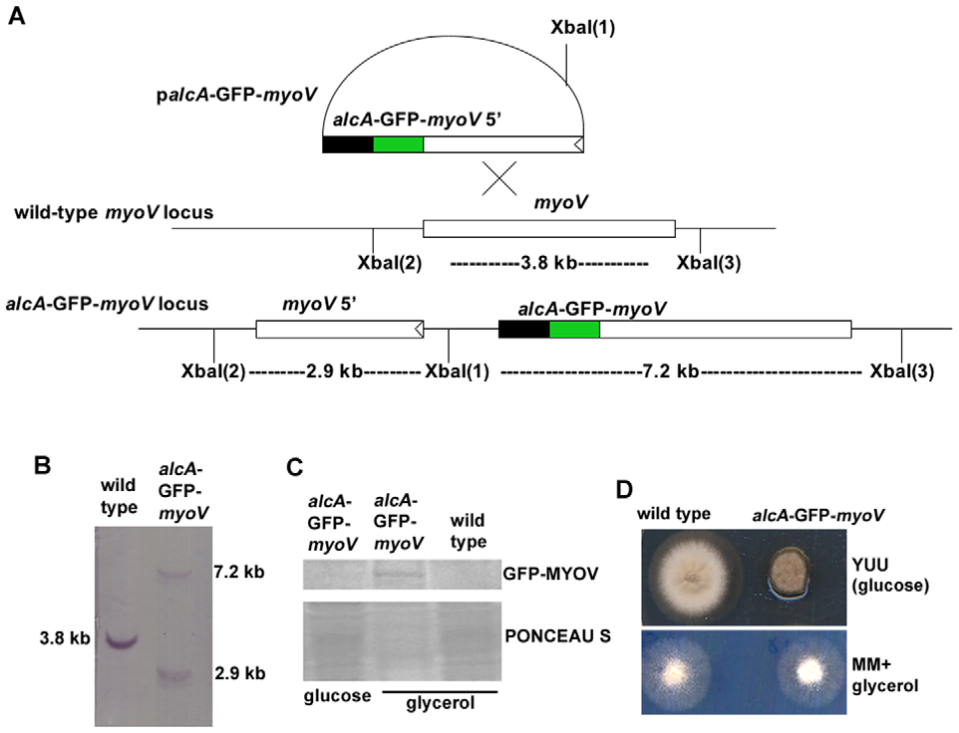
Construction of the *alcA*-GFP-*myoV* strain. (A) A diagram showing the homologous integration of the p*alcA*-GFP-*myoV* plasmid into the genome (see Materials and Methods for details). (B) A Southern blot confirming the homologous integration event. (C) A Western blot showing that the GFP-MYOV fusion protein can be detected in extracts of cells grown on glycerol but not on glucose. A protein extract from a wild type strain grown on glycerol was use as a negative control for the anti-GFP antibody. PONCEAU S staining of the same blot is shown as a loading control. (D) Growth phenotypes of the *alcA*-GFP-*myoV* strain grown on glucose (YUU) and glycerol (MM+glycerol) plates at 37°C for 2 days. The strains were point inoculated on different plates. Note that on YUU, the growth of the mutant is significantly reduced, but on MM+glycerol, the mutant colony is almost identical to a wild type colony.

We constructed *alcA*-GFP-*myoV*, a conditional null mutant of *myoV*, in which the *myoV* gene is replaced by a GFP-*myoV* fusion gene driven by the regulatable *alcA* promoter. Homologous integration of the GFP-tagged *myoV* sequence present in plasmid *palcA-GFP-myoV* into the genomic *myoV* locus generates two copies of *myoV*, a truncated *myoV* gene with its own promoter, and a full-length GFP-tagged *myoV* fusion gene under the control of the *alcA* promoter (Figure 1A). The homologous integration event was confirmed by a Southern blot analysis (Figure 1B). Moreover, the GFP-MYOV fusion protein is detectable by western blotting using anti-GFP antibody when the *alcA*-GFP-*myoV* cells are grown on glycerol, but not when glucose is used as a carbon source (Figure 1C). Shutting off *myoV* expression using glucose-containing YUU rich medium (called “MYOV depletion”) caused a noticeable reduction in colony size, with the diameter of *alcA*-GFP-myoV colonies being ∼50% that of wild type (Figure 1D). On non-repressive glycerol medium, the *alcA*-GFP-myoV strain grew as well as the wild-type strain (Figure 1D). This observation argues that the GFP-MYOV fusion protein is functional and that the phenotype on glucose-containing YUU medium is due to the depletion of MYOV rather than any dominant negative effect caused by the expression of the N-terminal portion of MYOV from the native promoter. Therefore, we used glucose-containing medium to examine the effect of MYOV depletion, and glycerol-containing medium to observe myosin-V localization in *A. nidulans*.

### GFP-MYOV localizes near the hyphal apex and septum

**Figure 2:**
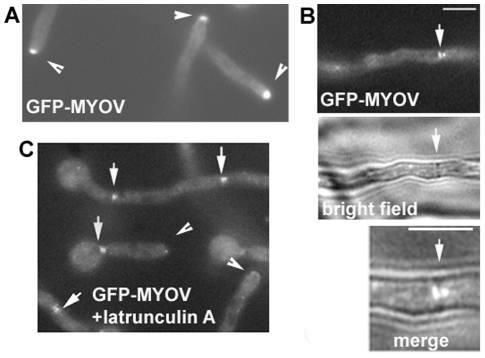
The localization of GFP-MYOV. (A) GFP-MYOV localizes to the hyphal tip. (B) GFP-MYOV localizes on two sides of the septum. (C) The effect of latrunculin A on GFP-MYOV localization. Cells were treated with 12 µM latrunculin A for 20 minutes. Arrowheads point to hyphal tips where the GFP-MYOV signals are diminished. Arrows point to GFP-MYOV signals near septa, which persist after treatments with latrunculin A. Bars, 5 µm.

Fluorescence microscopy revealed that GFP-MYOV was highly concentrated at the hyphal apex (Figure 2A). Given that exocytosis in *A. nidulans* most likely occurs at the hyphal apex [23], the concentration of MYOV in this region is consistent with it playing a key role in delivering secretory vesicles to support hyphal tip growth, as is the case for Myo2 in *S. cerevisiae* [24]. GFP-MYOV was also observed on both sides of the septum (Figure 2B), reminiscent of the localization of the Woronin body that plugs the septal pore [25]. The significance of this localization will be studied in the future. The accumulation of GFP-MYOV at the hyphal tip depends on the actin cytoskeleton, as short-term treatment with the actin-depolymerizing drug latrunculin A diminished the accumulation (Figure 2C). Interestingly, GFP-MYOV signals near septa were not affected by latrunculin A treatment (Figure 2C). While it is possible that the GFP-MYOV is not tethered at septa by the actin cytoskeleton, it is hard to rule out the possibility that some actin filaments at septa are resistant to latrunculin treatment.

### MYOV depletion inhibits hyphal elongation but not septation

**Figure 3:**
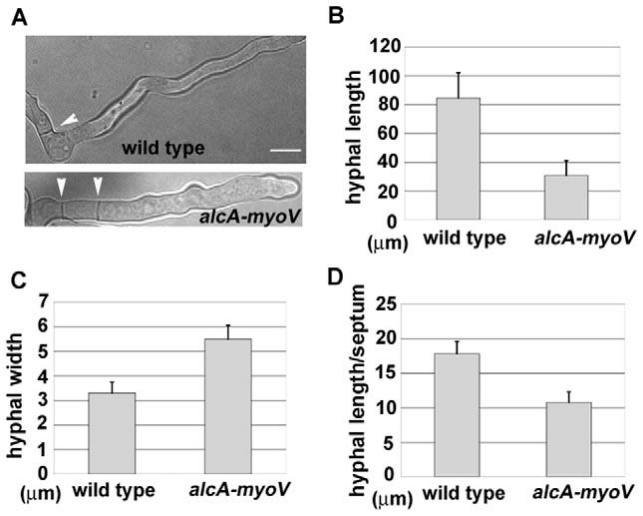
The growth phenotype of the *alcA-myoV* mutant. (A) Morphology of the *alcA-myoV* mutant 8 hours after germinating on repressive YUU medium at 37°C. A wild type cell is shown for comparison. Septa are indicated by arrow heads. (B, C and D) Quantitative analyses of hyphal length (B), hyphal width (C), and septum distribution (D) in wild type and the *alcA-myoV* mutant. Means and standard deviations are shown in the graphs. For hyphal length measurements, only those hyphae whose entire length could be seen through whole z-stacks (10–20 µm) were measured (n = 75 for the mutant; n = 70 for the wild type control). For hyphal width measurements, values were taken from hyphal regions where the hyphal width is uniform (n = 37 for both the mutant and the wild type control). For measurements of septum distribution, the lengths of the hyphal segments between adjacent septa were measured (n = 172 for the mutant; n = 147 for the wild type control). The differences between the mutant and the wild type controls are significant for hyphal length, hyphal width, and the length of hyphal segments (p<0.01 in all three cases). Bar, 5 µm.

While MYOV depletion in *A. nidulans* did not completely inhibit polarized growth, it did reduce the rate of hyphal growth significantly. Specifically, the size of *alcA*-GFP-*myoV* colonies on glucose-containing medium was ∼50% that of wild type cells (Figure 1D). During the course of this work, the Oakley lab made a deletion mutant of *myoV*. The colony size exhibited by their deletion mutant is almost identical to that of the *alcA*-based mutant described here (Dr. Berl Oakley, personal communication), confirming that *A. nidulans myoV* is indeed not essential. We performed a quantitative analysis on hyphal elongation rate and hyphal width for the *alcA-myoV* mutant. After incubation in glucose medium for 12 hours, the *alcA-myoV* mutant exhibited a dramatic difference in hyphal length relative to wild type cells (Figure 3A, B). Specifically, hyphae in the mutant were ∼3 times shorter that those in wild type cells (Figure 3B). Interestingly, myoV mutant hyphae were also ∼2 times wider than wild type hyphae (Figure 3A, C). Importantly, similar changes in morphology were also observed in the myosin-V deletion mutant (Dr. Berl Oakley, personal communication). Together, these results suggest that polarized growth at the hyphal tip is partially replaced by non-polarized cell expansion when MYOV is missing.

Septation was not abolished in the myoV mutant. Instead, septa in the mutant were spaced more closely as compared to wild type cells (Figure 3A, D). Specifically, the average length of hyphal segments, as defined by the distance between adjacent septa, was 17.8 µm in wild type cells, versus 10.7 µm in the myoV mutant (Figure 3D). Thus, although MYOV may transport certain components to the septum, as demonstrated by studies in *Schizosaccharomyces pombe* [26], this role is not essential for septum formation in *A. nidulans*. This result is consistent with an earlier observation made in *U. maydis* [27], indicating that myosin-V is not essential for septum formation in filamentous hyphae.

### MYOV supports significant polarized hyphal tip growth in the absence of microtubule-based transport

**Figure 4:**
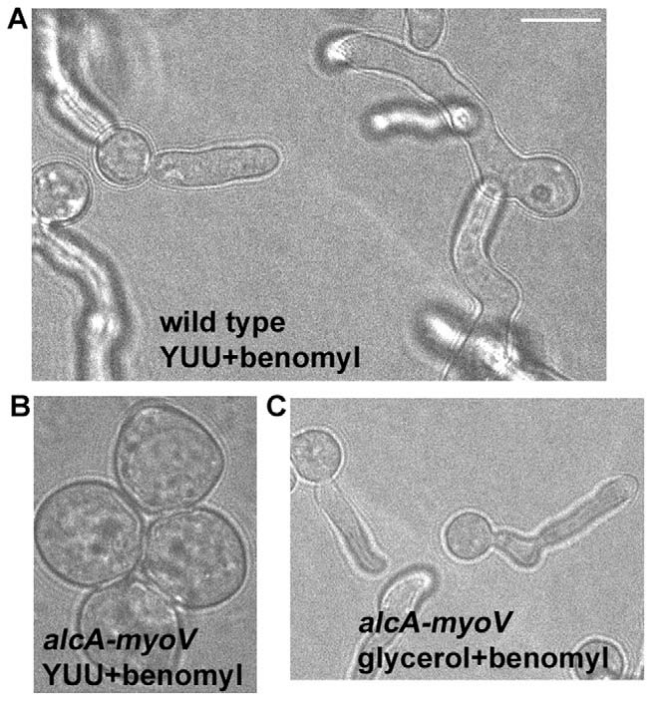
The *alcA-myoV* mutant loses polarity in the presence of benomyl when grown on repressive YUU medium. Cells were grown overnight at 32°C in YUU medium and in the presence of 2.4 µg/ml benomyl. Shown are a wild type strain (A) and the *alcA-myoV* mutant (B). The control in Panel C shows that the *alcA-myoV* mutant undergoes polarized growth when grown on glycerol medium in the presence of benomyl. All panels shown are at the same magnification. Bar, 5 µm.

We tested whether MYOV is essential for polarized hyphal growth in the absence of microtubules. After overnight treatment with benomyl to disassemble microtubules, wild type cells were still able to form short germ tubes (Figure 4A) [28]. Under the same conditions, MYOV depletion abolished polarized growth (Figure 4B). Thus, in the absence of microtubules, MYOV becomes essential for polarized growth. This result, together with similar results in *U. maydis* [21], strengthen the idea that efficient polarized growth in filamentous fungi requires a class V myosin and microtubule-based transport. That said, the data in Figure 4 does not rigorously exclude the possibility that MYOV only supports polarized growth in the absence of microtubules when cells are very short, since benomyl-treated cells fail to undergo long-distance hyphal tip extension [13], [23], [28].

**Figure 5:**
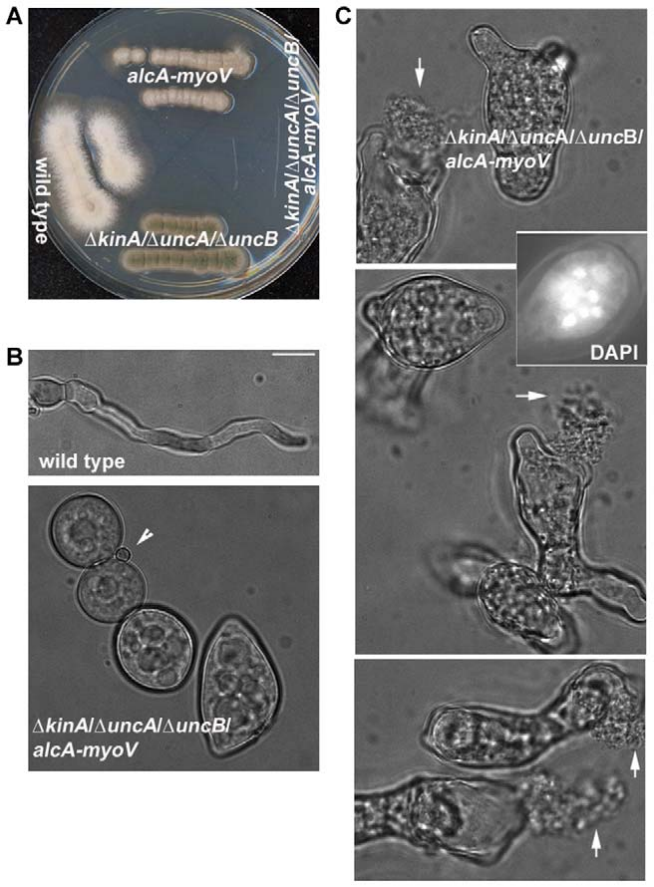
The *ΔkinA/ΔuncA*/*ΔuncB*/*alcA-myoV* quadruple mutant exhibits a severe polarity defect when grown on the repressive YUU medium. (A) The quadruple mutant failed to grow on a YUU plate. (B) In YUU liquid culture, the quadruple mutant failed to undergo polarized hyphal growth after an eight-hour incubation at 37°C. An arrow head points to a spore that had not undergone germination, thus indicating that mutant cells had undergone isotropic growth. (C) After an overnight incubation at 32°C, cells were highly abnormal in shape, and leakage of cell contents could be seen (arrows). An image of DAPI staining is included to demonstrate the presence of multiple nuclei. All panels shown are at the same magnification. Bar, 5 µm.

**Figure 6:**
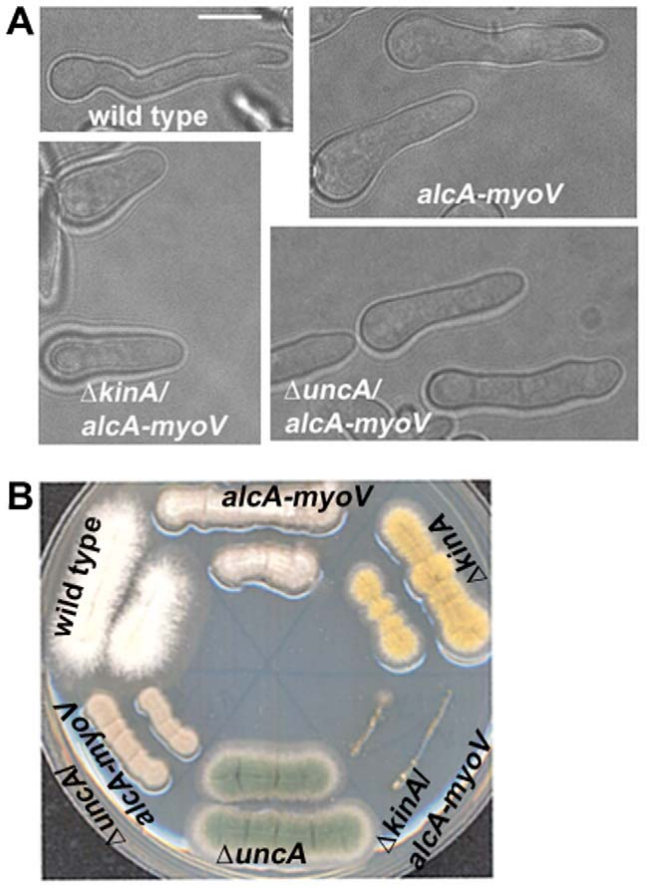
KINA is more critical than UNCA in the absence of MYOV. (A) Polarized growth occurs in both the Δ*uncA/alcA-myoV* and Δ*kinA/alcA-myoV* mutants. All strains were grown in YUU liquid medium for 6.5 hours at 37°C. All panels shown are at the same magnification. Bar, 5 µm. (B) After a 2-day incubation on a YUU plate at 37°C, the Δ*kinA*/*alcA-myoV* double mutant grew dramatically more slowly than either single mutant, while the growth of Δ*uncA*/*alcA-myoV* double mutant was only mildly worse than that of the *alcA-myoV* mutant.

To address the functional relationship between MYOV and microtubule-based transport in long hyphae where microtubules are distributed normally, we examined the effect of MYOV depletion in cells lacking kinesin-based transport. In most eukaryotic organisms, kinesin-1, kinesin-2 and kinesin-3 proteins transport membranous cargoes along microtubules to support a variety of cellular functions, including polarized secretion [29]–[31]. While kinesin-1 and kinesin-3 are both present in fungal genomes, kinesin-2 is not [32], [33]. In *A. nidulans*, one kinesin-1 gene, *kinA* [34], and two kinesin-3 genes, *uncA* and *uncB* [32], [35], have been identified. KINA is required for the microtubule-plus-end accumulation of cytoplasmic dynein and for hyphal growth [34], [36], [37]. UNCA is important for transporting vesicles towards the microtubule plus end [35], a function very similar to that of kinesin-3 in *U. maydis*, which powers early endosome transport towards the microtubule plus end [37], [38]. While the deletion mutant of *uncB* forms a normal colony, the deletion mutants of *uncA* and *kinA* produce small colonies [34], [35]. However, while the colony size of the Δ*uncA*/Δ*unc*B double mutant is similar to that of the Δ*uncA* mutant, the colony size of the Δ*uncA*/Δ*kinA* double mutant is similar to that of the Δ*kinA* single mutant (which is slightly smaller than the Δ*uncA* mutant) [35]. In this study, we constructed the triple kinesin null mutant Δ*kinA*/Δ*uncA*/Δ*uncB*. The growth of this triple kinesin null mutant is very similar to that of the Δ*kinA* single mutant, except that conidiation (asexual spore formation) in the triple mutant is slightly less robust than in the Δ*kinA* single mutant (Figure 5A, 6B).

To determine whether polarized growth still occurs when these three kinesins and MYOV are missing, we crossed the triple kinesin null mutant (containing the *ΔkinA*, *ΔuncA* and *ΔuncB* alleles) to the *alcA-myoV* mutant to obtain a quadruple mutant possessing all four mutant alleles. Importantly, while the quadruple mutant was able to grow and form colonies on glycerol medium, which allows expression of myoV, it was not able to grow on the glucose-containing medium, which represses myoV expression (Figure 5A). In terms of the terminal phenotype of the quadruple mutant grown in glucose medium, we found that it exhibited a severe polarity defect (Figure 5B, C). Specifically, after 8 hours of growth at 37°C, by which time wild type cells had formed elongated hyphae, the quadruple mutant exhibited a round morphology (Figure 5B). These mutant cells had clearly undergone isotropic growth since they were much larger in diameter than spores (Figure 5B, arrow head). After overnight incubation at 32°C, mutant cells exhibited dramatic defects in cell morphology (Figure 5C). This phenotype was also accompanied by leakage of cell contents, suggesting a cell wall abnormality (arrows in Figure 5C). While such a cell lysis phenotype is also exhibited by the *A. nidulans slaB* mutant, which is defective in endocytosis [39], the polarity defect exhibited by our quadruple mutant is much more severe. Indeed, many quadruple mutant cells were dead, as evidenced by a complete failure to undergo hyphal growth more than 15 hours after the cells had been shifted to glycerol medium. The lethality is almost certainly caused by the failure in polarized growth rather than a defect in mitosis, since the abnormally-shaped mutant cells did contain multiple nuclei (see insert in Figure 5C). Together, these results argue that MYOV is required in the triple-kinesin null mutant background to support the delivery of vesicular cargo needed for growth at the hyphal tip.

### KINA is crucial in supporting hyphal growth in MYOV-depleted cells

Unlike the quadruple mutant described above, introducing single mutant alleles of either KINA or UNCA into MYOV-depleted cells did not eliminate polarized growth (Figure 6A). However, colony growth of the Δ*kinA*/*alcA-myoV* double mutant on plates was dramatically slower than that of either single mutant (Figure 6B). In contrast, the growth of the Δ*uncA*/*alcA-myoV* double mutant was only mildly more attenuated than that of either single mutant (Figure 6B). Thus, loss of both UNCA and MYOV does not produce a much more dramatic growth defect than that caused by loss of MYOV or UNCA alone. These genetic results suggest that while MYOV and KINA function independently to a large extent in supporting hyphal growth, MYOV and UNCA may function in the same pathway to support hyphal growth, although it is not known whether UNCA and MYOV transport the same cargoes. That said, the functions of UNCA and MYOV are clearly not identical to each other, as MYOV is much more important than UNCA for supporting hyphal growth in the Δ*kinA* background. This conclusion is evident from the fact that the Δ*kinA/alcA-myoV* double mutant described in our current study is much sicker on plates than the Δ*kinA*/Δ*uncA* double mutant described previously, which looks very much like the Δ*kinA* single mutant [35].

## Discussion

In this study, we analyzed the function of myosin-V in the filamentous fungus *A. nidulans*, where both microtubules and the actin cytoskeleton contribute to the transport of organelles and vesicles required for polarized growth at the hyphal tip [13], [23], [40]–[42]. The results of our study indicate that MYOV is able to support significant hyphal elongation without microtubule-based transport. Importantly, our study also shows that plus-end-directed kinesin motors are able to support significant hyphal elongation without the aid of MYOV. Together, these observations, which mirror in part results obtained using *U. maydis* [16], [41], support the idea that myosin-V and plus-end-directed kinesin motors function in parallel to support fungal hyphal growth. The *A. nidulans* genome contains 11 kinesin-like genes [32]. Our current results demonstrate that, in the absence of MYOV, the loss of just three kinesin genes *kinA* (kinesin-1), *uncA* (kinesin-3) and *uncB* (kinesin-3) is sufficient to abolish polarized hyphal tip growth. Thus, none of the other plus-end-directed kinesins are able to transport the materials required for polarized growth at the hyphal tip in the quadruple mutant. Interestingly, both the Δ*kinA/alcA-myoV* and Δ*uncA/alcA-myoV* double mutants are able to undergo polarized growth initially, suggesting that kinesin-1 and kinesin-3 proteins compensate for each other in the absence of myosin-V. That said, the Δ*kinA/alcA-myoV* double mutant is much sicker on plates than the Δ*uncA/alcA-myoV* double mutant although the colony size of Δ*kinA* is similar or only slightly smaller than that of Δ*uncA*. Thus, KINA is more critical than UNCA for supporting hyphal growth in the absence of MYOV.

There are five myosin genes in *A. nidulans*, one coding for myosin-I [43], [44], one for myosin-II, one for myosin-V, and two for the Csm proteins in which a chitin synthase domain is fused to a myosin motor domain (myosin-17) [45], [46]. How these motors function together in hyphal tip growth and cytokinesis requires further study. While the exact cargoes of MYOV still need to be identified in *A. nidulans*, the results of this current study clearly demonstrate that MYOV is the main actin motor supporting hyphal tip growth. Moreover, our results show that this role becomes essential when microtubules and/or the plus-end-directed cargo-transporting kinesins are absent. Thus, while other myosin motors, such as the chitin-synthase-containing myosin, myosin-17, play a role in hyphal growth [45], [46], their functions must be either MYOV-dependent or microtubule-dependent since they fail to support hyphal growth in the absence of MYOV and microtubule motors. This idea is consistent with recent results demonstrating that myosin-17 in *U. maydis* is transported to the hyphal tip by myosin-V (myosin-5) and kinesin-1 along separate actin and microtubule tracks [41], [42].

In higher eukaryotic cells, myosin-V has been implicated in capturing cargoes at the periphery of the cell following their long-range, microtubule-dependent delivery [8]–[11], [47], [48]. Moreover, a direct physical interaction between myosin-V and kinesin [49] may serve to facilitate the switching of organelle movement from microtubules to actin tracks, as well as to enhance each motor's processivity [50]. Our current study does not exclude the possibility of microtubule-to-actin track switching as a transport mode for some cargoes when both the microtubule-based and the actin-based transport systems are functioning normally. However, in the absence of the microtubule motors implicated in transporting cargoes, myosin-V is clearly able to support some hyphal growth on its own. Thus, myosin-V appears able to transport cargoes in vivo in the absence of kinesin-mediated delivery of these cargoes to the microtubule plus end. This idea is consistent with the recent findings in *U. maydis*, *Dictyostelium*, and Purkinje neurons, that myosin-V can function in vivo as a point-to-point organelle transporter rather than simply as a cargo tether acting near the microtubule plus end [9], [41], [51].

The identity of the vesicular cargo(es) transported by myosin-V in *A. nidulans* is unknown. While the hyphal-tip localization of myosin-V and its importance in hyphal elongation are consistent with its function in transporting secretory vesicles as in *S. cerevisiae* and *Schizosaccharomyces pombe* [24], [26], [52], [53], the localization is also consistent with potential roles for the myosin in organizing the spitzenkörper, a vesicle-supply center [54]–[56], and/or in endocytosis at the hyphal tip region [23], [57]–[59]. In this study, we found that *A. nidulans* MYOV also localizes near septa. Indeed, the possibility that MYOV may play a role in secretion near septa cannot be excluded, since the hyphal tip may not be the only place where secretion occurs [60], [61]. Interestingly, the localization of MYOV near septa is confined to two spots on each side of the septum, which is almost identical to the localization of Woronin bodies [25], [62]. The Woronin body is a peroxisome-derived fungal organelle that plugs the septal pores to prevent the leakage of hyphal materials into a damaged hyphal segment [62]–[64]. Whether myosin-V is involved in Woronin body function will need to be determined in the future.

## Materials and Methods

### *A. nidulans* strains, growth conditions, and techniques

The strains used in this study are listed in Table 1. *Aspergillus nidulans* growth media, such as YAG, YUU, or MM+glycerol+supplements, growth conditions, DAPI staining of the nuclei, and *A. nidulans* molecular genetic methods were prepared or performed as described previously [65]. To repress myosin-V expression from the *alcA* promoter, the glucose-containing rich medium YUU was used. For benomyl treatment of cells, a final concentration of 2.4 µg/ml was used. For latrunculin A treatment of cells, a final concentration of 12 µM was used. Southern and western blot analyses were done as described previously [65]. The GFP antibody used in this study was from Covance.

**Table 1:** *A. nidulans* strains used in this work.

Strain name	Genotype	Source
GR5	*pyrG*89; *wA*3; *pyroA*4	G. S. May
R153	*wA*3; *pyroA*4	C. F. Roberts
ΔkinA or SNR7a	*ΔkinA::pyr4*; *yA*2; *pyroA*4	R. Fischer; (34)
ΔkinA (argB−)	*ΔkinA::pyr4*; *yA*2; Δ*argB*::*trpC*ΔB	R. Fischer; (34)
SNZ9	Δ*uncA*::*pyroA*; *pryG*89	(35)
alcA-myoV or C18	*alcA-GFP-myoV-pyr4; pyrG*89; *pyroA*4; *wA*3	This work
ΔkinA/alcA-myoV	*ΔkinA::pyr4; alcA-GFP-myoV-pyr4; yA2; possibly pyrG89; possibly pyroA4*	This work
XX210	Δ*uncA*::*pyroA*; *alcA-GFP-myoV-pyr4*	This work
RPA177	*ΔkinA::Afpyro; ΔuncA::AfpyrG; ΔuncB::bar; riboB2; pyroA4; pyrG89; ΔnkuA::Bar/argB+*	This work
TripleΔkinesins/alcA-myoV	*ΔkinA::Afpyro; ΔuncA::AfpyrG; ΔuncB::bar; alcA-GFP-myoV-pyr4; wA2; possibly ΔnkuA::Bar/argB+*	This work

### Construction of the *alcA*-GFP-*myoV* strain

We made a conditional null mutant of myosin-V, *alcA*-GFP-*myoV*, wherein the only functional copy of the *A. nidulans myoV* gene is under the control of the *alcA* promoter, which can be turned off by glucose and derepressed in glycerol medium. The strain was constructed as follows. Two oligonucleotides: myo5′Not1 (TCATGTGCGGCCGCTGGCGCATAATTATGAGGTCGGGACGAGGGCCTGG) and myos3′Sma1 (ACGACCCGGGCTTGTCGTCGAACATTATCTCAATGTACTTTCC), were used as primers to amplify from genomic DNA a fragment corresponding to the 5′ 935 bp of the myoV coding sequence. This PCR product was digested with NotI and SmaI and ligated into NotI/SmaI-digested pLB01 [66] creating plasmid p*alcA*-GFP-*myoV* in which the alcA promoter and the coding sequence for GFP are placed in frame immediately 5′ of the myoV heavy chain coding sequence. *palcA-GFP-myoV* was transformed into the *A. nidulans* strain GR5, transformants with similar growth defects were selected, and their genomic DNAs were subjected to a Southern blot analysis.

### Construction of the Δ*kinA*/Δ*uncA*/Δ*uncB* triple kinesin null mutant

The single kinesin null mutant was made using standard *A. nidulans* molecular genetic techniques. The coding sequences of *kinA*, *uncA* and *uncB* was replaced by the *A. fumigatus pyroA* gene, the *A. fumigatus pyrG* gene, and the glufosinate resistance gene (*bar*) of *Streptomyces hygroscopicus*, respectively [67], [68]. These gene replacement strategies were used in strains lacking *nkuA* [67]. The genotype of each kinesin null mutant was confirmed by PCR and Southern blot analyses. Genetic crosses were performed to create the triple kinesin null mutant. The genotype of the triple kinesin null was then confirmed by PCR.

### Introducing the kinesin null alleles into the *alcA-myoV* background

Genetic crosses were performed to create the Δ*kinA*/*alcA-myoV* and Δ*uncA*/*alcA-myoV* double mutants and the Δ*kinA*/Δ*uncA*/Δ*uncB*/*alcA-myoV* quadruple mutants. To obtain the double mutants, we selected progeny whose colony morphology resembled that of the Δ*kinA* or Δ*uncA* single mutants on glycerol plates, and then verified the presence of the *alcA-myoV* allele (which is actually *alcA*-GFP-*myoV*) by observing the hyphal tip accumulation of GFP-MYOV in cells grown on glycerol. To obtain the quadruple mutant, we selected the progeny whose colony morphology resembled that of the triple kinesin-null mutant on glycerol plates (which are similar in size to the Δ*kinA* single mutant) and that also contained the *alcA-myoV* allele, as evidenced by the hyphal-tip accumulation of GFP-MYOV in cells grown on glycerol. We then performed a genotyping analysis on 11 selected strains by PCR of the genomic DNA, and all five strains that were not viable on YUU were shown to contain the three kinesin-null alleles. This analysis confirmed the genotype of the quadruple mutants, and it also demonstrated that knocking out these three kinesins does not negatively affect the hyphal tip localization of GFP-MYOV.

### Image acquisition and analyses

Cells were grown in ΔTC3 culture dishes (Bioptechs, Butler, PA) containing 1.5 ml of MM medium containing glycerol (or glucose) and supplements. Images were captured as described previously [36] using an IX70 inverted fluorescence microscope (Olympus, Tokyo, Japan) (with a 100× objective) coupled to a 5-MHz MicroMax cooled charge-coupled device camera (Princeton Scientific Instruments, Monmouth Junction, NJ). IPLab software was used for image acquisition and analysis. For quantitative measurements of hyphal length, hyphal width, and septum distribution, we used a Zeiss confocal LSM510-meta with a 40× objective.

## Supporting Information

Figure S1 **Sequence comparisons among myosin V proteins from mouse and three fungal species.** Upper: Sequence alignments of the C-terminal cargo-binding domains of myosin-V proteins from *A. nidulans* (AmyoV), *U. maydis* (UmyoV), *S. cerevisiae* (Myo2p) and mouse (MmyoV). Bottom: A phylogenetic tree of four myosin V proteins from the above-mentioned species. The alignment and phylogenetic tree were made using the MegAlign tool of the DNA Star program.
(TIF)
